# Effect of Folic Acid, Betaine, Vitamin B_6_, and Vitamin B_12_ on Homocysteine and Dimethylglycine Levels in Middle-Aged Men Drinking White Wine

**DOI:** 10.3390/nu8010034

**Published:** 2016-01-12

**Authors:** Daniel Rajdl, Jaroslav Racek, Ladislav Trefil, Pavel Stehlik, Jana Dobra, Vaclav Babuska

**Affiliations:** 1 Institute of Clinical Biochemistry and Hematology, Faculty of Medical School and Faculty of Teaching Hospital, Charles University, 30460 Pilsen, Czech Republic; rajdl@fnplzen.cz (D.R.); racek@fnplzen.cz (J.R.), trefil@fnplzen.cz (L.T.); stehlikp@fnplzen.cz (P.S.); 2 Biomedical Center, Faculty of Medicine in Pilsen, Charles University in Prague, Alej Svobody 76, 32300 Pilsen, Czech Republic; 3 Department of Medical Chemistry and Biochemistry, Faculty of Medicine, Charles University, 30166 Pilsen, Czech Republic; jana.dobra@lfp.cuni.cz

**Keywords:** homocysteine, dimethylglycine, betaine, B-vitamins, moderate alcohol consumption

## Abstract

Moderate regular consumption of alcoholic beverages is believed to protect against atherosclerosis but can also increase homocysteine or dimethylglycine, which are putative risk factors for atherosclerosis. We aimed (1) to investigate the effect of alcohol consumption on vitamins and several metabolites involved in one-carbon metabolism; and (2) to find the most effective way of decreasing homocysteine during moderate alcohol consumption. Methods: Male volunteers (*n =* 117) were randomly divided into five groups: the wine-only group (control, 375 mL of white wine daily for one month) and four groups combining wine consumption with one of the supplemented substances (folic acid, betaine, and vitamins B_12_ or B_6_). Significant lowering of homocysteine concentration after the drinking period was found in subjects with concurrent folate and betaine supplementation. Vitamin B_12_ and vitamin B_6_ supplementation did not lead to a statistically significant change in homocysteine. According to a multiple linear regression model, the homocysteine change in the wine-only group was mainly determined by the interaction between the higher baseline homocysteine concentration and the change in dimethylglycine levels. Folate and betaine can attenuate possible adverse effects of moderate alcohol consumption. Dimethylglycine should be interpreted together with data on alcohol consumption and homocysteine concentration.

## 1. Introduction

**Figure 1:**
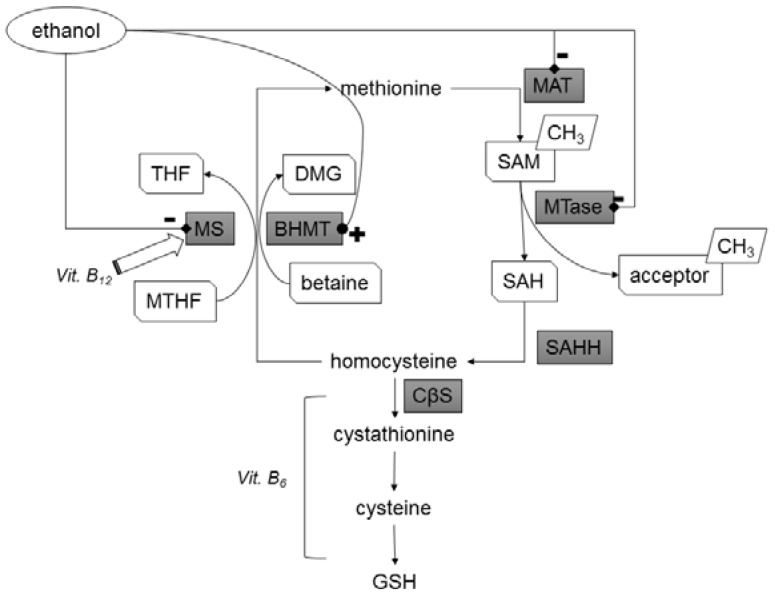
Effect of ethanol on key enzymes of one-carbon metabolism. Ethanol inhibits methionine synthase (MS), activates betaine homocysteine methyltransferase (BHMT), and possibly inhibits methionine adenosyltransferase (MAT) and several methyltransferases (MTase). Thus, the proposed effect of ethanol is a decrease in Hcy remethylation through the methionine synthase pathway, an increase in remethylation through the BHMT pathway, and a decrease in methylation potential through a decrease in SAM production and inhibition of methylation reactions [9].

Complications of atherosclerosis are leading causes of mortality and morbidity worldwide. Substances like folic acid, vitamins B_12_ and B_6_, or betaine (trimethylglycine) can influence the methionine-homocysteine cycle and thus change concentrations of homocysteine (Hcy) or dimethylglycine (DMG) [1], which are putative risk factors of atherosclerosis. High Hcy levels appear to be clearly associated with an increased risk of cardiovascular and cerebrovascular disease. However, Hcy does not appear to be as important as other risk factors, such as hypercholesterolemia, smoking, diabetes mellitus, and hypertension [2]. Despite promising results from observational studies (e.g., [3]), clinical trials have not confirmed efficiency of supplementation with low and high doses of folic acid and vitamins B_6_ or B_12_ in decreasing risk of cardiovascular diseases [4]_._ However, folic acid supplementation proved to be effective for stroke prevention [5,6,7]. On the other hand, there is still discussion of whether the clinical trials have the power to prove a potential benefit in a relatively short time and with concurrent hypolipidemic therapy (especially statins), and whether the complexity of influencing factors requires more detailed analysis [8]. One of the confounding factors is the consumption of alcoholic beverages. It is known that ethanol and its metabolites influence several key enzymes of the methionine-homocysteine cycle (Figure 1, e.g., they inhibit methionine synthase (MS), activate betaine homocysteine methyltransferase (BHMT), and possibly inhibit methionine adenosyltransferase (MAT)) and thus ethanol has a homocysteine-increasing effect, depletes liver S-adenosylmethionine (SAM), and causes fatty liver disease [9]. Due to the inhibition of methionine synthase, the BHMT pathway becomes more important as a source of SAM and a determinant of Hcy in alcohol consumers [10]. Therefore, in alcoholics, betaine theoretically seems to be a more effective methyl group donor than folate. To add more complexity, betaine may decrease the demand for choline methyl groups, thus increasing choline availability for lipid metabolism. Betaine can also support carnitine synthesis and thus a further lipotropic effect [11]. Furthermore, the transsulfuration pathway of Hcy degradation can be a source of cysteine and glutathione, which are major extracellular and intracellular antioxidants, respectively [8]. Of note, betaine and SAM supplementation increases the rate of ethanol elimination in rats [12].

Moreover, several clinical, epidemiological [13,14] and experimental studies [15] have proposed that light-to-moderate alcohol consumption is associated with a decreased risk of atherosclerosis. The relationship between alcohol and vascular risk or total mortality has been repeatedly depicted as a J-shaped curve. After an initial decrease in the vascular risk with increasing amounts of alcohol, the curve reaches a plateau and increases at higher doses [16,17,18]. Not only the amount of alcohol but also the drinking pattern is important, *i.e.*, protective effects are described in moderate regular drinkers, whereas episodic (binge) heavy drinking has detrimental effects [18]. Various mechanisms of action have been proposed for the manner in which moderate alcohol consumption affords its protective action. The reduced cardiovascular risk has been in turn explained by the ability of ethanol to increase plasma high-density lipoprotein-cholesterol (HDL) [19,20] and apolipoprotein A-I (apoA) [21], to decrease platelets aggregation and fibrinogen levels [22], and to promote antioxidant defenses [23,24]. It is still not clear whether a particular type of alcoholic beverage (red or white wine, beers or spirits) is important in this context [25,26,27,28] or not [29,30], and there is no consensus on the recommended amount (if any) of daily consumed alcohol [23,31,32]. However, it is obvious that alcohol consumption is one of the leading risk factors for mortality and morbidity worldwide [18], and any recommendation regarding positive effects of alcohol drinking must be managed extremely cautiously.

In summary, the interactions of ethanol metabolism with the methionine–homocysteine cycle, together with the effects of folic acid, betaine, and vitamins B_12_ and B_6_, are not fully understood and intervention trials are needed. We aimed (1) to investigate the effect of alcohol consumption on vitamins and several metabolites involved in one-carbon metabolism; and (2) to find the most effective way of decreasing Hcy during moderate alcohol consumption.

## 2. Materials and Methods

### 2.1. Study Subjects

One hundred and seventeen healthy middle-aged (37–65 years old) male participants were enrolled in this study. The selection was based on patients’ history (no chronic disease present, no medication, with exception of compensated hypertension treatment in 7 (6%) participants), laboratory results (alanine aminotransferase <1.4 µkat/L, amylase <1.7 µkat/L, triglycerides (TG) <3.0 mmol/L, creatinine-based estimated of glomerular filtration rate (according to Chronic Kidney Disease Epidemiology Collaboration (CKD-EPI) 2009 equation [33]) >1 mL/s and fasting serum glucose <6.2 mmol/L), basic clinical examination (blood pressure <140/90 mmHg), normal physical examination, and willingness to follow the study protocol. Participants were generally moderate alcohol consumers, and their alcohol consumption at the time of recruitment was 18.1 (9.3–29.9) g/day. Among the participants were 14 (12%) smokers. The highest achieved education was elementary education in 11 (9%), secondary education in 49 (42%), and tertiary education in 57 (49%) men.

All participants visited our outpatient department four times: the initial visit served for inclusion/exclusion of participants, visit one (visit 1) was after a month of abstinence from alcohol, visit two (visit 2) was after 1 month of daily white wine drinking and eventual supplementation, and finally visit three (visit 3) was after the second 1-month period of abstinence from alcohol. The period between visit1 and visit 2 and between visit 2 and visit 3 was 28 days. During each visit, participants underwent the following: measurement of blood pressure (automatic device Omron M5-I), body weight, and fat; a dialogue with emphasis on compliance and possible adverse effects of wine or supplemented substance; and fasting venous blood sampling (serum, heparin, citrate, and EDTA tubes; Vacuette Grainer, Kremsmuenster, Austria). Clinical characteristics of the study population are listed in Table 1.

**Table 1:** Basic characteristics of the study population. BP, blood pressure; eGFR, estimated glomerular filtration rate using the CKD-EPI 2009 creatinine formula [33]. The baseline characteristics were not significantly different between the experimental groups.

	Group	Visit 1	*p*	Visit 2	*p*	Visit 3
BMI (kg/m^2^)	All	26.0 (24.3–27.7)	1.000	26.3 (24.3–27.7)	**0.012**	26.1 (24.0–27.6)
	Control	25.5 (24.8–27.1)	1.000	25.6 (24.6–27.4)	0.231	26.0 (24.1–27.1)
	B_12_	25.6 (23.8–27.9)	1.000	25.5 (23.8–27.5)	0.502	25.6 (23.6–27.3)
	B_6_	25.1 (23.2–27.5)	1.000	25.1 (23.4–27.4)	0.072	25.0 (23.2–27.1)
	Betaine	26.4 (24.8–28.2)	1.000	26.7 (25.0–28.3)	1.000	26.6 (25.0–28.1)
	Folate	26.9 (25.5–29.3)	1.000	26.8 (25.2–29.2)	1.000	26.4 (25.7–29.1)
Body fat (%)	All	23.8 (20.7–27.0)	1.000	24.2 (20.6–27.3)	0.669	23.7 (21.1–26.8)
	Control	24.8 (22.4–26.9)	1.000	24.8 (21.0–27.2)	0.161	24.4 (19.9–26.5)
	B_12_	24.3 (20.4–27.2)	1.000	24.9 (19.5–27.5)	0.566	23.3 (19.4–26.2)
	B_6_	22.8 (20.4–24.4)	0.905	23.0 (20.8–25.3)	0.960	23.0 (21.4–25.0)
	Betaine	23.2 (20.7–26.2)	0.933	24.6 (20.6–27.3)	1.000	23.1 (21.6–27.2)
	Folate	25.9 (21.5–28.8)	1.000	25.8 (21.6–30.0)	1.000	25.9 (21.8–29.9)
Systolic BP (mm Hg)	All	128 (121–139)	1.000	129 (121–136)	0.362	130 (121–140)
	Control	127 (113–140)	0.453	131 (121–134)	1.000	131 (115–143)
	B_12_	126 (121–130)	0.770	124 (121–138)	1.000	128 (122–138)
	B_6_	128 (121–138)	0.297	129 (118–134)	1.000	128 (124–132)
	Betaine	135 (124–143)	0.161	130 (118–138)	0.078	138 (130–143)
	Folate	129 (120–141)	1.000	129 (115–143)	1.000	129 (120–138)
Diastolic BP (mm Hg)	All	82 (76–89)	1.000	82 (77–88)	1.000	83 (76–89)
	Control	81 (71–95)	1.000	84 (77–90)	1.000	83 (74–90)
	B_12_	80 (76–84)	1.000	82 (76–90)	1.000	81 (75–88)
	B_6_	84 (78–88)	1.000	81 (78–88)	0.782	81 (76–88)
	Betaine	86 (76–95)	0.676	81 (77–92)	1.000	87 (79–91)
	Folate	81.5 (78–87)	1.000	82 (79–87)	1.000	83 (75–88)
eGFR (mL/s)	All	1.31 (1.23–1.41)	**<0.0001**	1.37 (1.29–1.47)	0.116	1.35 (1.29–1.44)
	Control	1.32 (1.22–1.43)	1.000	1.33 (1.22–1.43)	1.000	1.34 (1.21–1.44)
	B_12_	1.28 (1.21–1.39)	0.461	1.34 (1.21–1.42)	0.426	1.33 (1.23–1.38)
	B_6_	1.37 (1.21–1.45)	**0.036**	1.40 (1.34–1.54)	0.295	1.37 (1.32–1.46)
	Betaine	1.33 (1.27–1.39)	**0.001**	1.37 (1.30–1.50)	1.000	1.40 (1.33–1.46)
	Folate	1.31 (1.24–1.44)	**0.003**	1.40 (1.34–1.50)	0.212	1.39 (1.29–1.48)

During the initial visit, the study protocol was explained to each participant, and they obtained a form to record possible non-adherence to study protocol. All participants were asked to not take any dietary supplements and to not change their lifestyle (except for the changes caused directly by the study protocol) during the study.

### 2.2. Study Design

All participants were randomly allocated into five approximately equally sized groups: four supplementation groups and one wine-only group. Each participant of the supplemented groups consumed 375 mL (*i.e*., 42 g of alcohol) of white wine *p.o.* daily in addition to one of following supplements: 40 mg of vitamin B_6_ *p.o.* (Pyridoxin Léčiva tablet) daily (*n =* 22); 200 µg of vitamin B_12_ *p.o.* (Nature's Bounty Vitamin B_12_ tablet) daily (*n =* 23); 5 mg of folic acid *p.o.* (Acidum Folicum Léčiva tablet) daily (*n =* 24); or 3 g of betaine *p.o.* (TMG, Life Extension, tablet) daily (*n =* 25). Participants in the wine-only group consumed only 375 mL of white wine *p.o.* daily (*n =* 23). The administered white wine was Müller Thurgau, produced by Vino Mikulov in 2004 (the basic characteristic of white wine used in our study is in Table 2). The study was approved by the Ethical Commission of University Hospital and Faculty of Medicine in Pilsen, and participants signed an informed consent. All blood samples were kept in the dark and cool box immediately after blood collection, and were centrifuged, processed, and frozen (−80 °C) within 1 hour of collection.

**Table 2:** Wine characteristics, including the content of alcohol and selected active substances. Müller Thurgau, produced by Vino Mikulov in 2004. Analysis was performed by High Performance Liquid Chromatography (HPLC).

Characteristics	
Alcohol Content (% vol.)	11.51
Polyphenolic compounds (mg/L)	268.7
Antioxidant Capacity—AOC (mmol/L)	4.18
Gallic acid (mg/L)	4.05
Catechin (mg/L)	9.0
Epicatechin (mg/L)	3.70
Resveratrol (mg/L)	0.19

We determined the levels of alanine aminotransferase (ALT; Dialab, Vienna, Austria), γ-glutamyl transferase (GGT; Human, Wiesbaden, Germany), a-amylase (DOT Diagnostics, Prague, Czech Republic), total cholesterol (TC; Human, Wiesbaden, Germany), HDL-cholesterol (HDL; Roche Diagnostics, Mannheim, Germany), triglycerides (TG; Human, Wiesbaden, Germany), apolipoproteins A and B (apoA and apoB; Tina-quant, Roche Diagnostics, Mannheim, Germany), hypersensitive CRP (hsCRP; Orion Diagnostica, Espoo, Finland), homocysteine (Hcy, enzymatic method from Carolina, Brea, CA, USA), uric acid (UA; DOT Diagnostics, Prague, Czech Republic), creatinine (Jaffé method, Olympus, Mishima, Japan), and glucose (Dialab, Vienna, Austria) in each serum sample with an Olympus AU 640 analyzer using the above-mentioned commercially available kits. LDL cholesterol was calculated according to Friedewald’s formula like TC minus HDL minus 0.45 × TG (all values in mmol/L, calculation was performed only when TG concentration was <4.5 mmol/L). Fibrinogen concentrations were assessed in citrate plasma with a CA-1500 analyzer (Sysmex, Japan) using a commercial set (Grifols DG-FIB, Barcelona, Spain). Serum levels of vitamin B_12_ and folic acid were determined by a chemiluminescent immunoassay with an Architect i2000 SR analyzer (Abbott, Chicago, IL, USA). For betaine (trimethyglycine) and DMG determination, we used a slightly modified HPLC method with UV detection of Laryea [34]. Serum level of vitamin B_6_ in its active form of pyridoxal-5′-phosphate (PLP) was determined by the HPLC method with fluorimetric detection of Talwar [35].

### 2.3. Statistical Analyses

Computations were performed with R 2.2.0 software (R Development Core Team 2004) and MedCalc for Windows, version 15.2.1 (MedCalc Software, Ostend, Belgium). Comparisons between values of samples from visit 1, visit 2, and visit 3 in the whole study group and among groups were performed using the two-way ANOVA with repeated measures. (One-way) ANOVA with repeated measures was used for detection of trends in separate intervention groups, for subsequent (post-hoc) pairwise comparisons, Bonferroni correction was used. The multiple linear regression model was built with Hcy change (visit 2 minus visit 1) as the dependent (explained) variable, and betaine change, DMG change, folate change, B_12_ change, baseline Hcy, and interaction of DMG change with baseline Hcy as the independent (explaining) variables. Unless stated otherwise, all data are presented as median (interquartile range).

## 3. Results

### 3.1. Concentration of Supplemented Substances

The baseline plasma concentrations of all supplemented substances were not significantly different between the experimental groups. Concentrations of all supplemented substances statistically significantly increased after supplementation in the appropriate groups (Table 3). In the wine-only group, there was an increase in betaine (*p =* 0.0016), and a decrease in vitamin B_12_ concentrations (*p =* 0.0001), whereas other vitamins remained unchanged (Table 3).

**Table 3:** Hcy and substances involved in its metabolism. Visit 1, after 1 month of alcohol abstinence; visit 2, after 1 month of white wine consumption; visit 3, after next month after visit 2 (2nd alcohol abstinence). PLP, pyridoxal-5-phosphate.

	Group	Visit 1	*p*	Visit 2	*p*	Visit 3
Hcy (µmol/L)	All	13.2 (11.7–15.6)	**0.017**	13.0 (10.9–14.6)	0.764	12.9 (11.4–14.8)
	Control	13.2 (11.7–16.0)	0.592	13.9 (12.9–15.4)	1.000	13.3 (12.6–15.8)
	B_12_	13.6 (11.9–17.6)	1.000	14.7 (12.2–15.7)	1.000	13.1 (12.3–15.3)
	B_6_	12.6 (11.6–14.7)	1.000	13.8 (10.9–14.9)	0.673	13.2 (11.2–14.8)
	Betaine	13.4 (11.8–14.6)	0.075	12.6 (10.4–13.3)	**0.039**	12.9 (11.7–13.8)
	Folate	13.0 (11.7–15.3)	**<0.0001**	10.8 (9.4–11.7)	0.123	10.9 (9.7–12.6)
B12 (ng/L)	Control	322 (243–374)	**0.0001**	275 (224–308)	1.000	300 (215–356)
	B_12_	291 (256–367)	**<0.0001**	439 (330–516)	**<0.0001**	297 (247–384)
	B_6_	316 (235–377)				
	Betaine	337 (290–372)				
	Folate	331 (281–397)				
PLP (nmol/L)	Control	13 (8.6–22.7)	0.616	16.8 (14.4–24.0)		
	B_6_	16.1 (11.9–20.7)	**<0.0001**	195.5 (144.0–241.5)		
Betaine (nmol/L)	Control	33 (29.0–48.0)	**0.002**	44 (34.0–51.0)		
	Betaine	33 (29.0–41.0)	**<0.0001**	159 (116.0–216.0)		
DMG (nmol/L)	Control	2.6 (1.73–3.10)	0.472	2.5 (1.30–3.40)		
	Betaine	2.1 (1.38–2.58)	**<0.0001**	10.7 (7.50–15.73)		
Folate (µg/L)	Control	4.2 (3.28–5.25)	0.075	3.9 (3.13–4.50)	0.540	4.1 (3.28–4.58)
	B_12_	5.8 (4.10–6.53)				
	B_6_	4.4 (3.80–6.70)				
	Betaine	5.1 (4.20–6.03)				
	Folate	4.8 (4.40–6.30)	**0.004**	20.9 (18.50–64.20)	**0.008**	8.75 (8.05–10.55)

### 3.2. Homocysteine Concentrations

**Figure 2:**
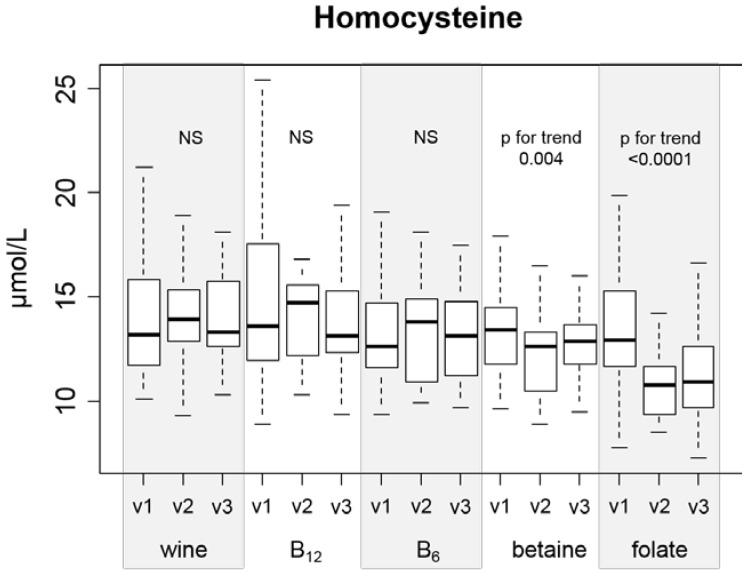
Serum total Hcy concentration in all groups, and visit 1 (v1), visit 2 (v2), and visit 3 (v3).

The baseline plasma Hcy concentration was not significantly different between the experimental groups. The change in Hcy levels in the wine-only group was not significant (*p =* 0.59, Table 3). A significant decrease in the Hcy concentration after the drinking period was found in subjects with concurrent folate supplementation (*p <* 0.0001, Table 3; *p* for quadratic trend <0.0001, Figure 2), and there was a significant quadratic trend in subjects with concurrent betaine supplementation (*p* for trend = 0.004). Vitamin B_12_ and vitamin B_6_ supplementation led to no statistically significant change in Hcy concentrations (Figure 2, Table 3). The effect of folate on Hcy lowering was statistically significantly greater than the effect of betaine (*p* = 0.001 for the difference in Hcy changes). All differences in Hcy concentrations are shown in Figure 2.

### 3.3. Determinants of Homocysteine Change

**Figure 3:**
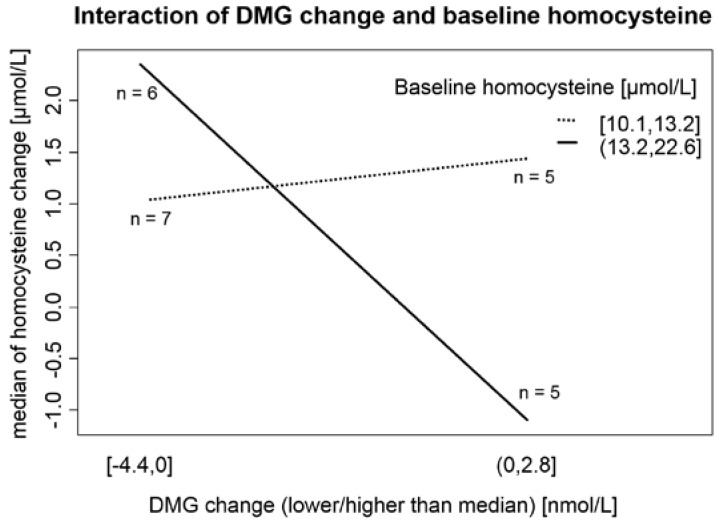
Interaction of DMG changes and baseline Hcy concentrations in relation to Hcy changes in the wine-only group after a month of drinking 375 mL of white wine daily. In participants with a baseline concentration of Hcy over its median (13.2 µmol/L), the change in Hcy levels after the wine-drinking period was significantly determined by the change in DMG, whereas in participants with lower baseline Hcy values, the BHMT system did not determine Hcy change.

According to the multiple linear regression model, the Hcy change in the wine-only group was mainly determined by the interaction between the higher baseline Hcy concentration and the DMG change. The details are explained in Table 4 and Figure 3, and are discussed below. Interestingly, DMG did not change in the wine-only group (*p =* 0.472), but DMG was significantly increased in the betaine group (*p <* 0.0001).

**Table 4:** Factors determining Hcy change during white wine drinking without vitamin supplementation (wine-only group). Adjusted *R*^2^: 0.62; *p =* 0.00095.

	Estimate	Std. Error	*p*
Intercept	1.2155	1.5784	0.4525
Betaine change	0.0307	0.0497	0.5456
Dimehylglycine change	1.9675	1.0412	0.0771
Folate change	−0.8404	0.4064	0.0552
B_12_ change	0.0162	0.0108	0.1531
Baseline homocysteine	−0.0472	0.1037	0.6558
Interaction of dimethylglycine change with baseline homocysteine	−0.2094	0.0668	**0.0064**

### 3.4. Selected Risk Factors of Atherosclerosis and Markers of Liver Damage

The effect of drinking white wine in all the groups on selected risk factors of atherosclerosis and markers of a potential toxic effect of alcohol are shown in Table 5. There was no statistically significant difference in these parameters between study groups (wine-only group and supplementation groups). However, we observed a significant increase in HDL (*p =* 0.009) and apoA (*p <* 0.0001) and a decrease in LDL (*p =* 0.0002) and fibrinogen (*p <* 0.0001) after a month of white wine consumption. There was a significant trend to decrease of apoB from visit 1 to visit 3 (*p* for trend < 0.001). On the other hand, there was a significant trend to increase TG and GGT after white wine consumption and a fall back in visit 3 (*p* for trend 0.0072 and 0.0017 resp.), while ALT and uric acid remained unchanged.

**Table 5:** Selected risk factors of atherosclerosis and markers of a potential toxic alcohol effect.

	Group	Visit 1	*p*	Visit 2	*p*	Visit 3
TC (mmol/L)	All	5.47 (4.95–6.27)	0.192	5.36 (4.96–6.14)	**0.0004**	5.26 (4.74–5.96)
	Control	5.65 (4.98–6.25)	1.000	5.51 (4.96–6.09)	**0.0005**	5.19 (4.68–5.75)
	B_12_	5.73 (5.18–6.01)	0.365	5.36 (4.98–5.97)	1.000	5.37 (4.85–6.10)
	B_6_	5.03 (4.80–6.19)	1.000	5.25 (5.06–5.52)	1.000	5.26 (4.68–6.00)
	Betaine	5.24 (4.93–6.28)	1.000	5.26 (4.90–6.30)	0.180	5.14 (4.66–6.24)
	Folate	5.49 (5.01–6.50)	0.387	5.40 (4.85–6.39)	0.064	5.18 (4.88–6.05)
HDL (mmol/L)	All	1.39 (1.23–1.56)	**0.0094**	1.43 (1.26–1.62)	**<0.0001**	1.35 (1.24–1.52)
	Control	1.31 (1.20–1.66)	0.108	1.42 (1.26–1.68)	**0.0003**	1.27 (1.16–1.46)
	B_12_	1.42 (1.24–1.56)	0.444	1.44 (1.26–1.68)	1.000	1.48 (1.33–1.59)
	B_6_	1.41 (1.33–1.46)	0.436	1.43 (1.33–1.58)	**0.015**	1.36 (1.27–1.48)
	Betaine	1.39 (1.25–1.61)	0.239	1.45 (1.32–1.59)	**0.0002**	1.34 (1.27–1.52)
	Folate	1.35 (1.22–1.56)	1.000	1.37 (1.16–1.53)	1.000	1.36 (1.24–1.48)
LDL (mmol/L)	All	3.55 (2.98–4.17)	**0.0002**	3.31 (2.90–3.89)	0.910	3.29 (2.85–3.84)
	Control	3.55 (3.01–4.15)	0.610	3.38 (3.06–3.90)	0.341	3.29 (3.09–3.70)
	B_12_	3.68 (3.14–4.15)	0.126	3.34 (3.00–3.81)	1.000	3.43 (2.98–3.70)
	B_6_	3.21 (3.01–4.15)	0.255	3.31 (2.86–3.64)	0.395	3.36 (2.89–4.01)
	Betaine	3.49 (2.97–4.18)	0.138	3.23 (2.73–4.01)	1.000	3.24 (2.74–4.08)
	Folate	3.55 (2.86–4.34)	0.292	3.27 (2.82–4.11)	0.190	3.12 (2.75–3.81)
ApoA (g/L)	All	1.27 (1.18–1.42)	**<0.0001**	1.38 (1.25–1.60)	**<0.0001**	1.29 (1.18–1.46)
	Control	1.25 (1.15–1.41)	**0.001**	1.34 (1.23–1.64)	**0.001**	1.29 (1.13–1.41)
	B_12_	1.32 (1.16–1.54)	**0.001**	1.38 (1.23–1.68)	0.397	1.39 (1.19–1.58)
	B_6_	1.28 (1.19–1.34)	**0.0003**	1.37 (1.27–1.53)	**0.001**	1.28 (1.20–1.37)
	Betaine	1.27 (1.21–1.40)	**0.002**	1.46 (1.31–1.52)	**0.001**	1.29 (1.22–1.46)
	Folate	1.29 (1.14–1.43)	**0.0001**	1.50 (1.26–1.60)	**0.002**	1.31 (1.16–1.43)
ApoB (g/L)	All	0.95 (0.82–1.13)	0.051	0.92 (0.82–1.10)	**0.031**	0.92 (0.80–1.06)
	Control	1.00 (0.82–1.13)	1.000	0.93 (0.84–1.00)	0.798	0.94 (0.85–0.98)
	B_12_	0.95 (0.86–1.09)	0.527	0.92 (0.83–1.07)	1.000	0.93 (0.82–1.04)
	B_6_	0.89 (0.82–1.10)	0.255	0.91 (0.79–0.99)	1.000	0.9 (0.78–1.05)
	Betaine	0.94 (0.80–1.11)	1.000	0.94 (0.82–1.14)	0.170	0.92 (0.76–1.08)
	Folate	0.98 (0.83–1.24)	0.740	0.94 (0.81–1.23)	**0.032**	0.91 (0.77–1.18)
TG (mmol/L)	All	1.15 (0.86–1.65)	0.234	1.25 (0.91–1.69)	**0.004**	1.10 (0.83–1.51)
	Control	1.14 (0.81–1.55)	0.579	1.11 (0.92–1.86)	**0.011**	1.10 (0.84–1.26)
	B_12_	1.11 (0.85–1.53)	1.000	1.09 (0.82–1.59)	1.000	0.97 (0.80–1.69)
	B_6_	1.04 (0.70–1.36)	0.165	1.06 (0.83–1.47)	0.187	0.93 (0.75–1.32)
	Betaine	1.13 (0.91–1.69)	0.578	1.38 (0.74–2.00)	0.612	1.05 (0.69–1.45)
	Folate	1.52 (1.12–2.00)	1.000	1.49 (1.24–2.02)	0.564	1.42 (1.05–1.91)
Fibrinogen (g/L)	All	2.62 (2.41–3.08)	**<0.0001**	2.51 (2.26–2.78)	0.135	2.55 (2.32–2.87)
	Control	2.62 (2.56–3.23)	**0.012**	2.45 (2.33–2.73)	1.000	2.51 (2.32–2.66)
	B_12_	2.69 (2.46–3.11)	**0.021**	2.55 (2.30–2.86)	0.215	2.62 (2.35–2.89)
	B_6_	2.73 (2.29–3.04)	**0.002**	2.25 (2.05–2.69)	0.129	2.55 (2.29–2.94)
	Betaine	2.62 (2.46–3.01)	0.761	2.48 (2.31–2.87)	1.000	2.51 (2.37–2.74)
	Folate	2.55 (2.38–2.90)	1.000	2.59 (2.32–2.90)	0.144	2.68 (2.55–2.99)
HsCRP (mg/L)	All	0.81 (0.54–1.75)	1.000	0.84 (0.50–1.68)	1.000	0.79 (0.46–1.60)
	Control	0.94 (0.48–4.24)	1.000	1.01 (0.46–1.81)	0.640	0.48 (0.37–1.32)
	B_12_	0.82 (0.60–1.57)	0.761	0.75 (0.39–1.63)	1.000	0.99 (0.51–1.54)
	B_6_	0.77 (0.53–1.37)	0.646	0.77 (0.45–1.23)	0.916	0.83 (0.51–1.51)
	Betaine	0.78 (0.56–1.81)	1.000	0.78 (0.36–1.47)	1.000	0.64 (0.45–1.63)
	Folate	0.78 (0.56–1.58)	0.374	1.08 (0.75–1.96)	0.411	0.94 (0.62–4.71)
ALT (µkat/L)	All	0.56 (0.43–0.73)	0.587	0.52 (0.45–0.68)	1.000	0.53 (0.42–0.72)
	Control	0.53 (0.43–0.73)	0.517	0.46 (0.42–0.61)	1.000	0.48 (0.39–0.62)
	B_12_	0.64 (0.49–0.78)	1.000	0.60 (0.50–0.79)	1.000	0.59 (0.45–0.77)
	B_6_	0.50 (0.36–0.65)	1.000	0.52 (0.45–0.66)	0.060	0.48 (0.38–0.63)
	Betaine	0.62 (0.45–0.82)	0.720	0.55 (0.45–0.68)	1.000	0.53 (0.49–0.82)
	Folate	0.54 (0.41–0.73)	1.000	0.49 (0.43–0.71)	0.500	0.57 (0.40–0.72)
GGT (µkat/L)	All	0.44 (0.30–0.59)	0.066	0.44 (0.33–0.65)	**0.001**	0.40 (0.32–0.62)
	Control	0.35 (0.30–0.53)	0.435	0.40 (0.29–0.53)	**0.009**	0.34 (0.26–0.47)
	B_12_	0.47 (0.36–0.68)	1.000	0.49 (0.39–0.72)	1.000	0.45 (0.35–0.68)
	B_6_	0.42 (0.25–0.54)	0.279	0.45 (0.30–0.61)	1.000	0.40 (0.26–0.54)
	Betaine	0.45 (0.31–0.57)	0.549	0.43 (0.33–0.64)	0.334	0.38 (0.32–0.61)
	Folate	0.53 (0.29–0.69)	**0.024**	0.57 (0.32–0.79)	0.172	0.56 (0.29–0.69)
Uric acid (µmol/L)	All	312 (264–351)	1.000	313 (269–350)	0.068	302 (250–337)
	Control	310 (261–346)	0.233	330 (277–364)	**0.003**	294 (250–327)
	B_12_	326 (265–360)	0.179	309 (258–348)	1.000	303 (258–322)
	B_6_	296 (266–326)	1.000	301 (254–344)	1.000	299 (250–335)
	Betaine	297 (265–364)	1.000	322 (284–348)	0.514	296 (262–336)
	Folate	316 (269–350)	1.000	306 (281–354)	1.000	311 (243–381)

## 4. Discussion

We present a randomized controlled trial that explores the interaction between moderate alcohol consumption, supplementation of substances important in the methionine methylation cycle, and risk factors of atherosclerosis.

There are two main findings in our study that deserve attention. First, folic acid and betaine are the only substances that can effectively lower Hcy during ingestion of a moderate amount (42 g daily) of ethanol (Figure 2). Second, the baseline Hcy value is important in the response of Hcy metabolism enzymatic systems to ethanol ingestion (Figure 3). To our knowledge, there is no published study with a similar design (concurrent controlled moderate alcohol consumption and supplementation of vitamins).

The observed effect of supplemented substances can be influenced by the selection of the dose of substance. Generally, we used similar vitamin doses to those commonly used in supplementation trials [36], although the folate dose was somewhat higher (5 mg daily). This dose was recommended by Brouwer [37] and was intentionally higher to overcome the effect of alcohol on Hcy levels. The ratio between the Recommended Daily Allowance (RDA; or in the case of betaine, mean average daily intake) and supplemented dose was 12.5 (5 mg/0.4 mg), 12 (3 g/0.25 g), 83 (200 µg/2.4 µg), and 26 (40 mg/1.5 mg) for folate, betaine, vitamin B_12_, and vitamin B_6_ respectively. We do not have dietary intake data for our participants, but the baseline concentrations of involved vitamins reflect intake of supplemented vitamins. The prevalence of presupplementation vitamin deficiency was low in the case of folate (no value <2 µg/L) and vitamin B_12_ (one value <110 ng/ L). However, in the case of vitamin B_6_ (PLP), the prevalence of plasma values <20 nmol/L (recommended cut-off for adequate intake [38]) was 71%, and 24% had values of <10 nmol/L. We have no adequate explanation for this unusually high prevalence of vitamin B_6_ deficiency in our study population.

We found a decrease in vitamin B_12_ and an increase in betaine after 1 month of drinking white wine (without any supplementation), while other vitamins (folate, PLP) remained unchanged. The data on the effect of moderate alcohol consumption on B-vitamin levels are conflicting in the literature. Van der Gaag observed in 11 healthy men drinking red wine, spirits, or beer (40 g of ethanol daily) a decrease in folate concentration only after drinking spirits, with no change in B_12_ in all study groups, and an increase in vitamin B_6_ after drinking beer and, surprisingly, red wine and spirits too [39]. Gibson showed a decrease in folate and vitamin B_12_ after drinking red wine or spirits (24 g of ethanol daily) in 78 healthy males [40]. Laufer demonstrated a decrease in vitamin B_12_ and no change in folate in 52 postmenopausal women receiving 15 and 30 g daily (ethanol in orange juice) in a diet-controlled crossover trial [41]. Although our study was not diet-controlled, we can reasonably suppose that eating habits of our participants did not change substantially, thus interference of ethanol (white wine) on the absorption process of vitamin B_12_ is the most probable cause. The observed increase in betaine concentration has not been reported in any published study. However, Mar [42] showed that red and white wines have small amounts of betaine, and we can hypothesize that this could be a reason for the increase.

The effect of folic acid and betaine on Hcy levels in different groups of healthy subjects or patients is well described in the literature [3,4,11]. In our setting (concurrent alcohol consumption), folate and betaine were the only supplemented substances that effectively decreased Hcy, with folate being the most effective. Therefore, we did not confirm the proposed (due to the metabolic interference of ethanol with folate metabolism that was discussed in the Introduction) superiority of betaine. As discussed above, selection of the supplement dose could be the reason for this observation. Our data did not show efficacy of either vitamin B_6_ or vitamin B_12_ supplementation in decreasing Hcy. This is in concordance with other authors [37], but van der Gaag [39] found a correlation between B_6_ and Hcy change, which was especially pronounced after beer consumption. This does not directly contradict our results, as beer is a source of folic acid, and it is known that a combination of folic acid and vitamin B_6_ is more effective than folic acid itself [43]. Together with the relatively high supplemented doses of vitamins B_6_ and B_12_ (relative to RDA), our results underscore the fact that vitamins B_12_ and B_6_ are not effective as Hcy-lowering agents in this setting.

The hypothesis that even mild alcohol consumption is associated with a total Hcy increase is supported by several studies [39,40], including our previous research [44]. The fact that some studies (and this article) did not show an increase in Hcy after moderate alcohol consumption [45,46] indicates that influencing factors are rather complex. However, the effects of ethanol on several enzymes involved in Hcy metabolism (Figure 1) and the consistent finding of hyperhomocysteinemia in alcoholics [47] allows us to consider ethanol as a generally hyperhomocysteinemic substance.

In our study, 27% (*n =* 31) of subjects can be classified as having moderate hyperhomocysteinemia (concentration of Hcy >15.0 mmol/L) according to presupplementation values. Prevalence of mild hyperhomocysteinemia is a relatively common finding and ranges from a comparable 24% in Greater Tunis [48] to 68% in northern China [49]. Plasma Hcy concentration reflects a complex status of remethylation and transsulfuration pathways (including levels of folate, vitamin B_12_, betaine, and vitamin B_6_). Furthermore, plasma DMG levels are a better marker for the amount of remethylation in the BHMT system than plasma betaine concentration [11]. Our results suggest that in individuals with higher Hcy (>13.2 µmol/L), the main factor that governs the Hcy change after consumption of 42 g of ethanol daily is DMG (as a marker of Hcy remethylation to methionine mediated by BHMT; Figure 2). This unique finding from our interventional trial can partially explain the conflicting results of studies observing the association between alcohol consumption and Hcy levels (moderate alcohol consumption as a factor associated with lower plasma Hcy levels in Hordaland study [50] *vs.* alcohol consumption associated with increased plasma Hcy levels [44,49,51]). Svingen *et al.* [1] found in a large (4150 patients) prospective study that high plasma DMG levels enhance the risk of acute myocardial infarction. Unfortunately, the authors did not mention alcohol consumption as a possible confounding factor and 80% of participants were on statin therapy (usually in addition to other drugs, factors known to influence Hcy and DMG levels [52,53]). On the other hand, our participants were not undergoing statin or fibrate therapy, and we obtained fasting morning serum samples, thus allowing more controlled and standardized results. Therefore, our results may change the interpretation of DMG as a putative risk factor of atherosclerotic complications.

The results of our study on lipoprotein particles are consistent with various data in the literature [20]: in the whole study group (regardless of supplementation groups), HDL-cholesterol plus apoA increased, and LDL cholesterols plus apoB and coagulation factor fibrinogen decreased after a month of white wine drinking. There was no statistically significant difference between study groups, and a specific effect of betaine on lipoprotein levels could not be demonstrated. Betaine supplementation showed a lipotropic effect in some studies [11], and betaine is used in animal breeding to increase lean body mass and in humans to prevent alcoholic [54] and non-alcoholic steatohepatitis [55]. The mechanism of this action is not clear, but methylation of active substances (e.g., norepinephrine to epinephrine [54], synthesis of carnitine [11] or synthesis of creatine [56]) and methylation of DNA and subsequent regulation of gene expression (e.g., increased apoB synthesis [57], activation of peroxisome proliferator-activated receptor-α (PPARα), or an increase in microsomal triglyceride transfer protein [58]) are probably involved. Sparks [57] depicted, in an animal model (rats), a rise in apoB mRNA expression after BHMT activation and betaine supplementation that led to increased VLDL and TG production and a decrease in TG in liver tissue. On the other hand, Wang [58] found other mechanisms of betaine protection against steatohepatitis, *i.e.*, prevention of increased expression of enzymes involved in fatty acid synthesis (fatty acid synthase, acyl-CoA oxidase) and prevention of the PPARα and microsomal triglyceride transfer protein mRNA increase, which are factors involved in lipoprotein metabolism and fatty acid breakdown. Interestingly, *apoB* expression was not influenced by betaine. CRP in concentrations below 10 mg/L (hsCRP) can be used for atherosclerosis risk assessment. Moreover, a J-shaped association between hsCRP and alcohol consumption is described [59]. One of explanations of this phenomenon is that low alcohol concentrations may inhibit interleukin-6 secretion from adipocytes [60] and folate can also modify this relation [61]. We observed a significant positive correlation between initial hsCRP (visist 1) and BMI (*r* = 0.45, *p <* 0.0001). In the regression model with initial hsCRP as independent and initial BMI, body fat and pre-study alcohol consumption as independent variables, BMI and alcohol consumption were marginally significant (*p =* 0.055 and 0.056 resp.; adjusted *R*^2^ = 0.19, *p* < 0.0001; data not shown). There were no significant changes of hsCRP in all supplemented groups (Table 5); therefore, it is not reasonable to seek for a relationship to white wine and supplemented substance administration.

We conclude that the effect of consumed white wine on lipoproteins is “atheroprotective” (decrease in the LDL/HDL ratio) and decreases coagulation by lowering fibrinogen. On the other hand, known “side effects” (increase of liver enzymes, triglycerides and uric acid) of alcohol consumption were not (in our setting) clinically significant: TG and GGT slightly (statistically significantly) increased and ALT remained unchanged. Alcohol consumption can cause fatty liver disease (alcoholic fatty liver disease, AFLD), similarly, obesity, insulin resistance and other conditions are associated with nonalcoholic liver disease (NAFLD) [62]. In our study, we have no diagnostic measurement (e.g., liver biopsy or ultrasound) to evaluate prevalence of AFLD or NAFLD and possible effect of alcohol consumption and supplemented substances on these entities. Diagnostic performance (AFLD, NAFLD) of laboratory tests and BMI are very limited, however, there were 85 (73%) participants with BMI ≥ 25 kg/m^2^ and 19 (16%) participants with BMI ≥ 30 kg/m^2^. In addition, 15 participants (12%) had fasting glucose ≥5.6 mmol/L. Thus overweight, obesity and possibly insulin resistance are prevalent in our study population and presence of NAFLD cannot be excluded. A closer look to individual values of ALT, GGT and TG as possible laboratory surrogates for AFLD and NAFLD (Table 6) reveals that especially increased TG are prevalent in our study population.

**Table 6:** Number (percentage) of participants with values of ALT, GGT or TG higher than upper reference limit.

	Visit 1	Visit 2
ALT >1 µkat/L	10 (9%)	9 (8%)
GGT >1.3 µkat/L	2 (2%)	3 (3%)
TG >1.7 mmol/L	26 (22%)	29 (25%)

Some authors [63] describe a relationship between activity of GGT and ALT. To further elucidate factors influencing changes of ALT, GGT and TG, we built multiple regression models with changes (before and after white wine drinking) of ALT, GGT and TG as dependent variables and starting value (visit 1) of ALT, GGT, TG, BMI, body fat, type of supplemented substance and initial (pre-study) ethanol consumption as explaining variables. Generally, the most important factor influencing changes in abovementioned markers are the starting values of it (e.g., the higher the concentration of TG was before white wine drinking, the lower the increase after white wine drinking, Table 7). Type of supplemented substance did not influence changes in these markers, thus none of the supplemented substances can be considered as “hepatoprotective” in this setting. Some authors [64] published indirect evidence that modest alcohol consumption (<10 g/day) can protect against NAFLD. In our study, none of the laboratory markers were influenced by the pre-study consumption of ethanol. However, there was a one-month abstinence from ethanol before visit 1; therefore, a putative effect of modest alcohol consumption could be diminished.

**Table 7:** Factors influencing changes of TG, GGT and ALT. Ethanol, pre-study ethanol consumption in grams per day.

	TG Change			GGT Change			ALT Change		
	Estimate	Std. Error	*p*	Estimate	Std. Error	*p*	Estimate	Std. Error	*p*
Intercept	1.0451	0.4488	**0.0219**	0.0741	0.0855	0.3885	0.3548	0.1588	**0.0277**
BMI (kg/m^2^)	−0.0491	0.0248	0.0502	−0.0051	0.0047	0.2795	−0.0144	0.0087	0.1016
TG (mmol/L)	−0.3359	0.0928	**0.0005**	−0.0091	0.0182	0.6188	−0.0551	0.0336	0.1036
Body fat (%)	0.0406	0.0181	**0.0269**	0.0066	0.0034	0.0562	0.0117	0.0063	0.0669
ALT (µkat/L)	−0.2276	0.1697	0.1827	−0.0397	0.0461	0.3906	−0.4328	0.0847	**<0.0001**
GGT (µkat/L)	−0.0278	0.2133	0.8966	−0.1421	0.0520	**0.0075**	0.0207	0.0798	0.7956
Group B_12_	−0.2247	0.1641	0.1740	−0.0237	0.0316	0.4559	0.0576	0.0577	0.3205
Group B_6_	−0.2127	0.1643	0.1983	0.0178	0.0313	0.5703	0.0223	0.0576	0.7003
Group betain	−0.0357	0.1640	0.8282	0.0304	0.0314	0.3354	0.0480	0.0581	0.4105
Group folate	−0.1381	0.1635	0.4002	0.0578	0.0317	0.0714	0.0549	0.0581	0.3467
Ethanol (g/day)	0.0053	0.0030	0.0797	0.0006	0.0006	0.3102	0.0010	0.0011	0.3579
	*R*^2^ = 0.1214; *p =* 0.0095			*R*^2^ = 0.1273; *p =* 0.0095			*R*^2^ = 0.2659; *p* < 0.0001		

The liver plays a central role in production and catabolism of Hcy and there is some data that Hcy is higher in patients with NAFLD, [65] but, in our study, we found no correlation between putative markers of NAFLD (ALT, GGT, TG, BMI) and Hcy (neither in absolute values before white wine drinking, nor comparing changes of these markers before and after wine consumption period; data not shown).

The main limitation of our study is the availability of DMG measurements in the wine-only group and in the betaine-supplementation group. Concentration of DMG was not measured in folate, vitamin B_12_ and B_6_ groups. This fact does not allow us to derive conclusions about DMG changes in relation to ingestion of other supplemented substances. Another limitation of our study is that we did not determine genetic factors (e.g., MTHFR mutations) that clearly influence Hcy levels (one-carbon metabolism). Finally, one of the important limitations of our study is that the intervention and follow-up times were short, thus not allowing us to concentrate on the relationship between biochemical markers and outcome (mortality and morbidity) of study subjects.

## 5. Conclusions

In summary, folate and betaine are the most promising substances that can attenuate possible adverse effects of moderate alcohol consumption. DMG as a putative risk factor of atherosclerotic complications should be interpreted together with data on alcohol consumption and Hcy concentration.

## Abbreviations

The following abbreviations are used in this manuscript:

DMG	Dimethylglycine
Hcy	Homocysteine
MS	Methionine synthase
BHMT	Activate betaine homocysteine methyltransferase
MAT	Methionine adenosyltransferase
SAM	*S*-adenosylmethionine
HDL	High-density lipoprotein
LDL, VLDL	Low-density lipoprotein, Very-low-density lipoprotein
apoA, apoB	Apolipoprotein A-I, Apolipoprotein B
TG	Triglycerides
CKD-EPI	Chronic Kidney Disease Epidemiology Collaboration
EDTA	Ethylenediaminetetraacetic acid
eGFR	Estimated glomerular filtration rate
ALT	Alanine aminotransferase
GGT	γ-glutamyl transferase
TC	Total cholesterol
hsCRP	Hypersensitive C-reactive protein
UA	Uric acid
PLP	Pyridoxal-5′-phosphate
RDA	Recommended Daily Allowance
PPARα	Peroxisome proliferator-activated receptor-α
MTHFR	Methylene tetrahydrofolate reductase
AFLD	Alcoholic fatty liver disease
NAFLD	Nonalcoholic fatty liver disease
